# Pharmacological ablation of the airway smooth muscle layer—Mathematical predictions of functional improvement in asthma

**DOI:** 10.14814/phy2.14451

**Published:** 2020-06-12

**Authors:** Graham M. Donovan, Kimberley C. W. Wang, Danial Shamsuddin, Tracy S. Mann, Peter J. Henry, Alexander N. Larcombe, Peter B. Noble

**Affiliations:** ^1^ Department of Mathematics University of Auckland Auckland New Zealand; ^2^ School of Human Sciences The University of Western Australia Crawley WA Australia; ^3^ Respiratory Environmental Health Telethon Kids Institute The University of Western Australia Nedlands WA Australia; ^4^ School of Biomedical Sciences The University of Western Australia Crawley WA Australia; ^5^ School of Public Health Curtin University Bentley WA Australia

**Keywords:** airway hyper‐responsiveness, asthma

## Abstract

Airway smooth muscle (ASM) plays a major role in acute airway narrowing and reducing ASM thickness is expected to attenuate airway hyper‐responsiveness and disease burden. There are two therapeutic approaches to reduce ASM thickness: (a) a direct approach, targeting specific airways, best exemplified by bronchial thermoplasty (BT), which delivers radiofrequency energy to the airway via bronchoscope; and (b) a pharmacological approach, targeting airways more broadly. An example of the less well‐established pharmacological approach is the calcium‐channel blocker gallopamil which in a clinical trial effectively reduced ASM thickness; other agents may act similarly. In view of established anti‐proliferative properties of the macrolide antibiotic azithromycin, we examined its effects in naive mice and report a reduction in ASM thickness of 29% (*p* < .01). We further considered the potential functional implications of this finding, if it were to extend to humans, by way of a mathematical model of lung function in asthmatic patients which has previously been used to understand the mechanistic action of BT. Predictions show that pharmacological reduction of ASM in all airways of this magnitude would reduce ventilation heterogeneity in asthma, and produce a therapeutic benefit similar to BT. Moreover there are differences in the expected response depending on disease severity, with the pharmacological approach exceeding the benefits provided by BT in more severe disease. Findings provide further proof of concept that pharmacological targeting of ASM thickness will be beneficial and may be facilitated by azithromycin, revealing a new mode of action of an existing agent in respiratory medicine.

## Graphical abstract

Airway smooth muscle (ASM) plays a major role in acute airway narrowing and reducing ASM thickness should be considered more frequently by researchers as a therapeutic target in asthma: reduction in ASM thickness is expected to attenuate airway hyper‐responsiveness and reduce disease burden. Two approaches to targeting ASM are: (a) a direct approach, targeting specific airways, best exemplified by bronchial thermoplasty (BT), which delivers radiofrequency energy to the airway via bronchoscope; and (b) a pharmacological approach, targeting all airways equally. An example of the less well‐established pharmacological approach is the calcium‐channel blocker gallopamil which in a clinical trial effectively reduced ASM thickness; other agents may act similarly. In view of established anti‐proliferative properties of the macrolide antibiotic azithromycin, we examined its effects in a mouse model and report a reduction in ASM thickness of 29% (*p* < .01). We considered the potential functional implications of this finding, if it were to extend to humans, by way of a mathematical model of lung function in asthmatic patients which has previously been used to understand the mechanistic action of BT. Predictions show that pharmacological reduction of ASM in all airways of this magnitude would reduce ventilation heterogeneity in asthma, and produce a therapeutic benefit similar to BT. Moreover there are differences in the expected response depending on disease severity, with the pharmacological approach exceeding the benefits provided by BT in more severe disease. Findings provide further proof of concept that pharmacological targeting of ASM thickness will be beneficial and may be facilitated by azithromycin, revealing a new mode of action of an existing agent in respiratory medicine.

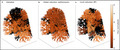

## 1 INTRODUCTION

Contraction of airway smooth muscle (ASM) drives acute airway narrowing in asthma (King, Pare, and Seow 1999). As such, reducing the thickness of the ASM layer is a promising therapy which is yet to be fully exploited. Two distinct modalities to reducing ASM thickness are the pharmacological approach, such as the calcium channel blocker gallopamil, which in a clinical trial appeared to reduce ASM mass (Girodet et al., 2015), or more local approaches like bronchial thermoplasty (BT), in which specific (large) airways are directly targeted using radiofrequency energy delivered via bronchoscope (Castro et al., 2010; Cox, Miller, McWilliams, Fitzgerald, & Lam, 2006; Cox et al., 2007; Pavord et al., 2007). There are notable differences between these approaches. While the degree of ASM reduction achieved by gallopamil was less than BT (gallopamil 20% compared with as much as 75% for BT), oral administration allows therapeutic processes to take place throughout the bronchial tree. In comparison, BT is largely confined to a local area of treatment and this confinement has generated controversy regarding the mechanism of action of BT (Donovan, Elliot, Green, James, and Noble 2018).

In this study, we address several pertinent questions. Firstly, we trialled the capacity of another clinically relevant pharmacological agent, the macrolide antibiotic azithromycin, to reduce ASM mass. Azithromycin was chosen due to its anti‐proliferative properties, demonstrated in ASM cultures in vitro (Stamatiou et al., 2009), and its role in reducing asthma exacerbations (Hiles, McDonald, Guilhermino, Brusselle, & Gibson, 2019). However, the exact mechanism of action has been the subject of much research and remains unclear (Parnham et al., 2014). We here show that in naive mice, azithromycin reduces ASM thickness, and by a similar magnitude to gallopamil (Girodet et al., 2015). These data help to clarify azithromycin's mechanism of action in asthma: that the ASM reduction occurs in naïve mice supports an inflammation‐independent pathway. It also allows us to add azithromycin to the existing work on gallopamil, providing further evidence that pharmacological ablation of the ASM is a viable approach.

Following on from this finding, we used an established mathematical model (Donovan, 2016, 2017; Donovan et al., 2018) to consider the effects of potential pharmacological reduction of ASM in asthma. That is, if the finding that azithromycin reduces ASM mass in naïve mice does extend to asthma in humans, what would the expected consequences of this be? To this end we examined, by way of the model, the relative efficacy of a treatment which globally reduces ASM (pharmacological approach), but by a lesser degree, as opposed to one with a larger effect on ASM but confined to a smaller number of airways (i.e., BT). Such modeling has been successful in helping to understand the underlying mechanism of action of BT by considering not just the treatment effects on individual airways, but also the ways in which the alteration of one airway can affect its neighbors, both via flow coupling and airway‐parenchymal interdependence (Donovan, Elliot, Boser, et al., 2019; Donovan et al., 2018; Donovan, Elliot, Green, James, & Noble, 2019; Langton, Noble, Thien, & Donovan, 2019). In this way we are able to compare the potential benefits of pharmacological therapies, which reduce ASM throughout the bronchial tree, with localized therapies, such as BT, in terms of their relative efficacy and the specific characteristics of the treatment response.

Overall, the present study combines data from an azithromycin mouse model, demonstrating ASM reduction in vivo, with a theoretical approach, using the mathematical model to project the consequences of similar reductions in patients with asthma. Results indicate that azithromycin does reduce ASM thickness and may be at least as effective as BT.

## 2 METHODS

### 2.1 Azithromycin mouse model

Adult (9‐week‐old) male BALB/c mice were obtained from the Animal Resources Centre and housed in a pathogen‐free environment at the Telethon Kids Institute. Mice were weighed daily, maintained under a 12‐hr light/dark cycle and given access to food and water ad libitum. The study maintained strict compliance to the National Health and Medical Research Council's Australian code for the care and use of animals for scientific purposes (8th edition, 2013). All protocols were approved by the Telethon Kids Institute Animal Ethics Committee (Application #339) and The University of Western Australia Animal Ethics Committee (RA/3/100/1576).

Mice were randomized to receive s.c injection of azithromycin or saline (control) for 11 consecutive days (day 0 to day 10). The dose of azithromycin (50 mg/kg) was based on the findings of Beigelman et al. (2009). On day 11, mice were euthanized by a two‐step process. Following sedation with methoxyflurane (Medical Developments International Ltd, Springvale, Australia), euthanasia was achieved by barbiturate overdose (sodium pentobarbitone, 160 mg/kg by i.p injection).

### 2.2 Airway morphometry and histology

Bronchoalveolar lavage fluid was initially collected for a separate study; lungs were subsequently fixed by intra‐tracheal instillation of 4% formaldehyde at a transrespiratory pressure of 10 cmH_2_O (Hsia et al., 2010). The left lobe was embedded in paraffin with the proximal end facing down. Two sections from the central bronchus were stained using hematoxylin & eosin (Wang et al., 2018). The first section was acquired just below the transition from extra‐ to intra‐parenchymal bronchus (middle region in (Sato, Bartolak‐Suki, Parameswaran, Hamakawa, & Suki, 2015)), and the second section marginally deeper into the lung (toward the lower region in (Sato et al., 2015)). These regions are hereafter referred to as “Proximal” and “Distal,” respectively. The cross‐sectional area of ASM layer and internal perimeter of the basement membrane (P_bm_) were measured using the Stereo Investigator software (version 10, MBF Bioscience, Vermont, USA). The square roots of areas were then corrected by the P_bm_, the standard index of airway size (James, Hogg, Dunn, & Pare, 1988). Measurements were performed by a blinded observer (KCWW).

Analyses were conducted using two‐way ANOVA with treatment (saline vs. azithromycin) and location (proximal vs. distal) as repeat measures factors (SigmaPlot version 13.0, Systat Software Inc).

### 2.3 Mathematical model

To consider the potential functional consequences of such a reduction of ASM in humans, we adapted a previously developed mathematical model of BT (Donovan et al., 2018) to the context of global reduction of ASM by pharmacological means. In brief, the model approach is as follows: the airway tree structure is generated using a statistical model fit to extensive structural airway data. This dataset consists of 1,515 individual airways from *n* = 57 donors, and further broken down into non‐fatal asthma (NFA, *n* = 32) and fatal asthma (NA, *n* = 25). Subject characteristics are given in Table 1. Formalin fixed lung sections from whole left lungs were acquired from the Prairie Provinces Fatal Asthma Study (Tough, Green, Paul, Wigle, & Butt, 1996) and analyzed as described previously (Donovan et al., 2018). The measurements of these airways, in terms of P_bm_, wall area, ASM area, and anatomical level, are used to inform a statistical model of airway structure at each airway order (Horsfield, 1990). In this way, simulated lung structures of different disease severity can be generated by Monte Carlo simulation (Mooney, 1997).

**TABLE 1:** Subject characteristics from (Donovan et al., 2018; Tough et al., 1996)

	Nonfatal asthma (*n* = 32)	Fatal asthma (*n* = 25)
Age, years, mean ± *SD*	35 ± 11	33 ± 14
Gender, male/female	16/16	15/10
Inhaled/oral corticosteroid use, number (%)^a^	9 (53)	12 (92)^†^
Ever smoked, number (%)^a^	11 (52)	9 (64)
Perimeter of the basement membrane, mm, mean ± *SD* (range)	11.3 ± 1.6 (1.7–30.8)	11.8 ± 1.6 (1.8–32.5)
Body mass index, mean ± *SD* (range)^a^	32 ± 8 (16–45)	26 ± 6 (15–41)*
Age at onset of asthma, years, median (IQR)^a^	17 (10–26)	9 (3–39)
Duration of asthma, years, median (IQR)^a^	17 (9–21)	17 (7–22)
Asthma severity, “Severe”, number (%)^a^	8 (38)	9 (64)

^a^ Incomplete data set.

* *p* < .05 versus Nonfatal asthma.

^†^ *p* = .04

**FIGURE 1:**
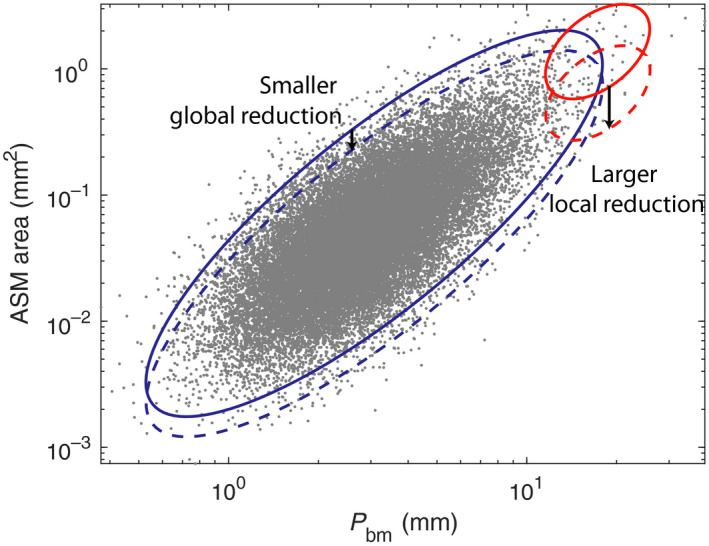
Schematic of low level global ASM reduction (pharmacological; blue) compared with more substantial local ASM reduction (BT; red). Perimeter of epithelial basement membrane is used as an index of airway size. Elliptical visual aids indicate the airways before (closed line) and after (dotted line) each treatment modality

Within these structures, we calculated the functional flow patterns using the model developed in (Donovan, 2016, 2017) (based on (Anafi & Wilson, 2001; Leary, Winkler, Braune, & Maksym, 2014; Venegas et al., 2005)), and subsequently calculated the impedance (including resistance) using a *post‐hoc* approach (Lutchen & Gillis, 1997; Thorpe & Bates, 1997). In particular, we use the resistance at 5 hz, which is known to correlate with other well‐known measures such as FEV_1_ (Akamatsu et al., 2017; Vink, Arets, van der Laag, & van der Ent, 2003). This is done first in the pre‐treatment control for both NFA and FA. The underlying airway structure is then altered according to the treatment protocol; either a 75% reduction in ASM mass in the BT‐treated airways only, as per (Donovan et al., 2018), or a uniform reduction in the ASM in all airways. See Figure 1 for a schematic illustration of the imposed reductions in ASM for each treatment in the model. In the case of pharmacological global treatment, we allowed a variable level of reduction to account for potential variations in pharmacological effectiveness, assuming either 10%, 20%, or 30% reduction in ASM mass in all airways. In all cases, 20 independent simulations were performed, at varying doses of contractile agonist, and we assessed the treatment response of that simulated cohort.

Ventilation heterogeneity is characterized using the spatial heterogeneity index (SHI), introduced in (Donovan et al., 2018). This assessed the heterogeneity of the flow using an equally weighted combination of the coefficient of variation and the spatial correlation, the latter using Moran's I statistic. It attempts to capture both the overall heterogeneity of the flow, but also the extent to which this heterogeneity is spatially structured (e.g., clustered ventilation defects).

## 3 RESULTS

**FIGURE 2:**
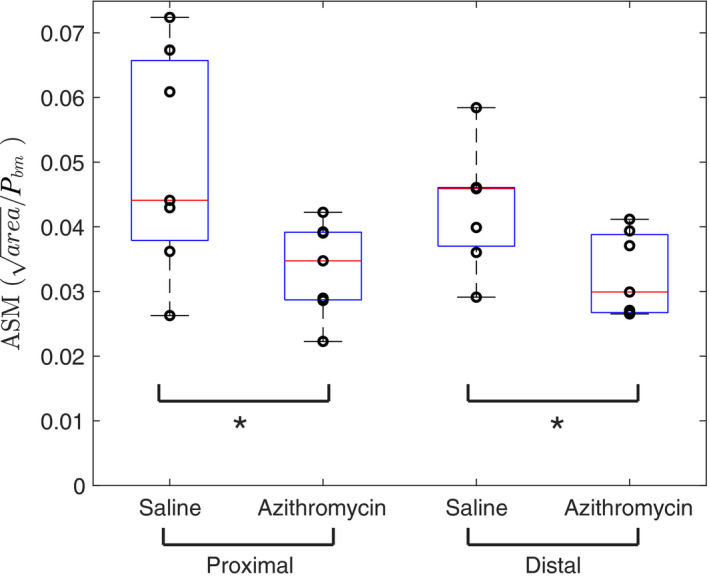
Azithromycin reduces ASM thickness in both proximal and distal locations compared with control (saline) mice. Area of ASM is normalized to P_bm_. *Statistical significance at *p* < .05. *N* = 7 for each group, individual data points shown superimposed on box plots

Mice treated with azithromycin (50 mg/kg for 11 days) had reduced ASM compared with control (saline) mice; see Figure 2. This effect was present at both proximal and distal locations. Testing by repeated measures two‐way ANOVA revealed a significant effect of azithromycin (*p* = .007) with no effect of location (*p* = .670) and no interaction (*p* = .834). The average ASM reduction across both locations was 29%.

**FIGURE 3:**
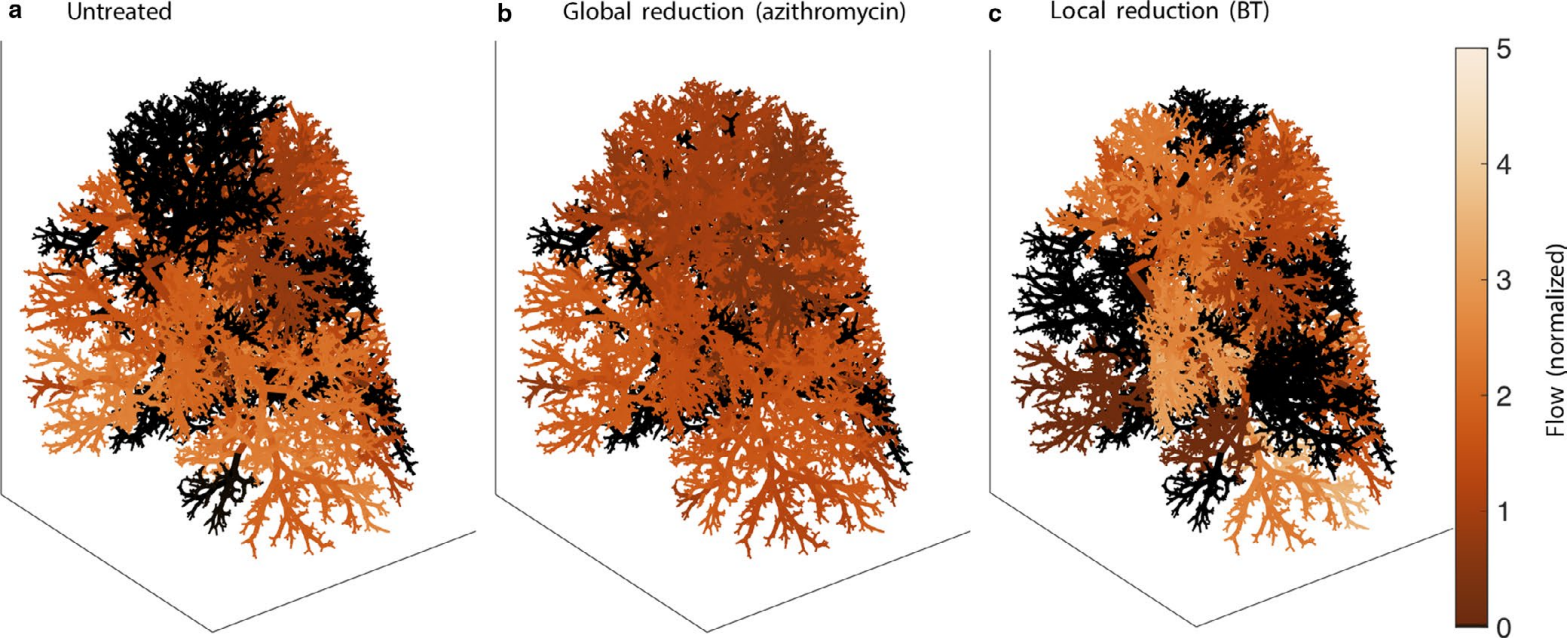
Example flow patterns in single FA case at approx. 75% of maximal ASM activation. (a) untreated; (b) global 30% reduction in ASM (azithromycin); (c) local 75% reduction in ASM in treated airways (BT). Flow is normalized to nominal (Donovan, 2017)

To assess the effects of such a reduction in asthma, simulations of the mathematical model were performed assuming different levels of global ASM reduction as a result of theoretical pharmacological treatment: 10%, 20%, or 30% reductions. These were compared with both untreated simulations, with no structural changes, and simulated BT, with a 75% ASM reduction in the BT‐treated airways only. A sample simulation set is shown in Figure 3, with the left‐hand panel (a) being untreated, the center panel (b) showing the effects of a 30% global reduction in ASM, and the right‐hand panel (c) showing the effects of simulated BT. In both cases, the flow patterns have clearly been altered by treatment, and in different ways.

**FIGURE 4:**
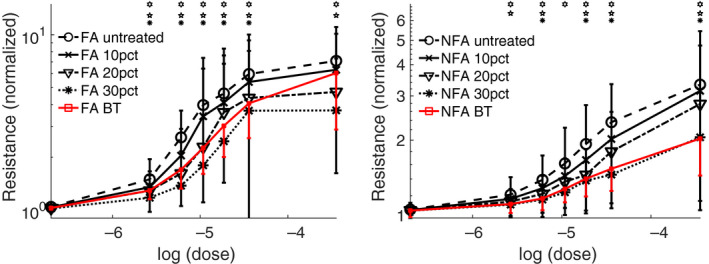
Simulated dose‐response curves comparing global reduction in ASM at various levels (10%, 20%, 30%) with BT’s 75% reduction in ASM in the treated central airways only. Left panel: fatal asthma (FA). Right panel: non‐fatal asthma (NFA). Note that the vertical scales are log scales, and differ between the two panels. Error bars are standard error. *,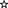,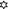 = statistical significance at *p* < .05 by paired *t*‐test for untreated versus 10%, 20%, and 30% reduction, respectively. For clarity of comparison, resistance values are normalized to a reference value at zero ASM activation for each simulated patient at baseline

Aggregating statistics over the entire simulated cohort, for both NFA and FA, and for varying doses of contractile agonist (e.g., methacholine), yields the dose‐response curves shown in Figure 4. The left‐hand panel is for FA, while the right‐hand panel is for NFA. In both cases, the expected pattern of progressive decreases in resistance with further reduction in ASM is apparent. What is also apparent is the characteristic lack of response at baseline, compared with the clear separation between untreated and treated curves as agonist is increased, previously seen in BT (Donovan et al., 2018). The simulated BT dose‐response curves are also overlaid (in red) for easy comparison. Note the difference in relative response between FA and NFA; in the former case, BT is approximately equivalent to a 20% reduction in ASM, while in the latter, BT is almost exactly equivalent to a 30% reduction in ASM. This difference will be considered further in the discussion. The out‐of‐phase component of the impedance (elastance or reactance) displays qualitatively similar results (data not shown), and an estimate of the aggregate of small airway resistance shows an approximately 10% shift in favor of the global ASM reduction relative to BT.

**FIGURE 5:**
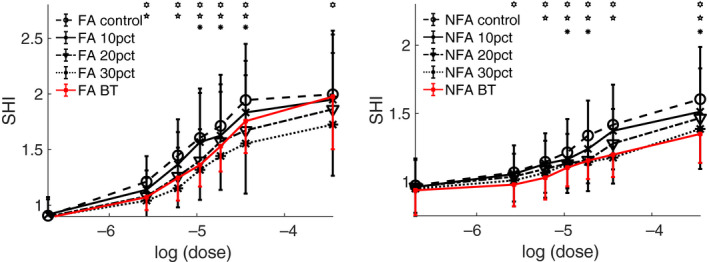
Response of the spatial heterogeneity index (SHI) of flow as contractile agonist is varied. Details as in Figure 4 except that a linear scale is used for the vertical axis

The changes to the ventilation heterogeneity can be more directly quantified using the spatial heterogeneity index (SHI), which is constructed so as to reflect not just the overall heterogeneity of the flow, but also the extent to which it is spatially structured (see methods). The SHIs for the simulated cohort are shown in Figure 5, using the same presentation style as Figure 4. The same basic conclusion is apparent: in FA, BT is approximately equivalent to a 20% global reduction in ASM, albeit with a sensitivity shift, while in NFA the equivalence is closer to 30% global reduction.

## 4 DISCUSSIONS AND CONCLUSIONS

This study has two key findings: first, the macrolide antibiotic azithromycin induces a significant reduction of ASM thickness in naive mice of almost 30%. Second, we consider, by way of a mathematical model, the potential functional consequences of such a global reduction in ASM in humans, and find that global ASM reduction of this magnitude has similar functional improvement to that of BT (Donovan et al., 2018). Namely, a reduction in ventilation heterogeneity, with minimal changes in resistance at baseline, but clear attenuation of bronchoconstriction to increasing ASM stimulation, consistent with studies reporting reduced airway hyperresponsiveness in asthmatic patients after azithromyocin (Ekici, Ekici, & Erdemoglu, 2002; Piacentini et al., 2007). These findings demonstrate the potential of pharmacological‐induced ablation of the ASM layer as well advancing our understanding of the mechanism by which azithromycin reduces asthma exacerbations. Specifically, since azithromycin induced a notable reduction in ASM in *naïve* mice, as opposed to an allergen sensitized model, therapeutic action is independent of inflammation.

Azithromycin is a macrolide antibiotic that appears to have antibacterial, antiviral and anti‐inflammatory properties. Due to these potential benefits, multiple clinical trials have been run regarding its efficacy in reducing asthma exacerbations (Gibson et al., 2017, 2019; Johnston et al., 2016) and a recent meta‐analysis concluded that maintenance use of azithromycin reduces exacerbations in patients with asthma, including eosinophilic, non‐eosinophilic, and severe asthma subtypes (Hiles et al., 2019). The mechanism of action by which it does so, however, has been unclear. The present results suggest that reduction in ASM thickness is a plausible mechanism of action for azithromycin's role in reducing exacerbations in asthma, and moreover offers a possible explanation for the lack of consistently demonstrable improvement in conventional measures of lung function such as FEV_1_ (Reiter et al., 2013), discussed below.

In terms of a direct effect on the structure of the ASM layer, azithromycin exhibits anti‐proliferative (Stamatiou et al., 2009) and autophagic properties (Stamatiou, Boukas, Paraskeva, Molyvdas, & Hatziefthimiou, 2010), the latter of which may lead to apoptosis. Ongoing proliferation of ASM cells occurs even in non‐asthmatic subjects (James et al., 2018; Stamatiou et al., 2010) such that any attenuation in cell proliferation (or increased apoptosis) should reduce ASM thickness. Importantly, ASM cell cultures describe above (Stamatiou et al., 2009, 2010) were maintained under non‐inflammatory conditions, entirely consistent with our present data that azithromycin decreases ASM thickness in naïve mice and hence via an inflammation‐independent mechanism. Azithromycin's mode of action is therefore quite diverse and in addition to anti‐proliferative actions on the ASM, its range of effects includes direct relaxation of pre‐contracted ASM (Daenas, Hatziefthimiou, Gourgoulianis, & Molyvdas, 2006; Massey et al., 1988), reduced inflammation and goblet cell hyperplasia (Beigelman et al., 2009; Kang et al., 2016) and maintenance of normal epithelial barrier function (Slater et al., 2016). None of these additional effects of azithromycin were considered in the present study which was designed to assess the functional consequences of reduced ASM thickness and to compare local changes in the large airways to more globally distributed changes in muscle mass.

If we were to assume that azithromycin's principal mechanism of action is reduction in ASM thickness (for analytical purposes only, acknowledging the drug's diverse mode of action), then the functional improvements accruing to this structural change may be difficult to detect at baseline, but instead only manifest in situations in which a higher level of ASM activation is present (see Figures 4 and 5, with little‐to‐no change at baseline, but progressively greater improvement with increasing dose). For safety reasons, these situations are not often apparent in the clinical laboratory, and hence changes of this sort are relatively difficult to detect using conventional measures of lung function (FEV_1_ etc.). These more severe situations do still occur outside of the clinical testing environment, and so would be expected to manifest in reported exacerbations (as observed with azithromycin), ACQ/AQLQ scores, and similar measurements.

The differential response to simulated pharmacological treatment depending on asthma severity is interesting. In FA, a 20% global reduction in ASM is approximately equivalent to the 75% ASM reduction in the BT‐treated central airways. Changes are most apparent at the top of the dose response curve, but there is also a possible shift in sensitivity. In NFA, on the other hand, a 30% global reduction is almost exactly equivalent to BT, with no sensitivity shift. This difference between NFA and FA responses reflects the structural differences between the two groups, with the latter exhibiting not just greater ASM on average, but also increased heterogeneity (Donovan et al., 2018). The implication is that a pharmacological approach, with uniform reduction, could be relatively favored in more severe disease, while a local approach such as BT might be favored in relatively less severe disease. All factors being equal, treating physicians may be more inclined to prescribe oral administration of a pharmaceutical which would avoid sedation or general anesthesia and acute injury effects associated with BT (Castro et al., 2010; Cox et al., 2007; Pavord et al., 2007).

Much of the motivation for the present study was generated due to recent findings that gallopamil, in the context of a clinical trial, reduces ASM in severe asthma (Girodet et al., 2015). This provided a proof‐of‐concept that pharmacological agents could be used to target airway remodeling, in a relatively non‐invasive manner compared with BT. The gallopamil study has, however, not been without controversy due to the manner in which ASM thickness was assessed (Sumino, Sheshadri, & Castro, 2015) and we note that gallopamil may directly attenuate ASM contraction (Daenas et al., 2006; Massey et al., 1988). Nonetheless, the present finding that azithromycin has a similar effect, in mice, to that claimed for gallopamil in humans, strengthens the rationale for pharmacological agents as a means of effecting global reduction of ASM. The subsequent modelling is not specific to azithromycin or gallopamil, but rather to a generic reduction in ASM of the specified level by any (likely pharmacological) means.

Similarly, the assumed level of 75% reduction in ASM in the BT treated airways, though well within the range of reported reductions in biopsy studies, has been questioned (Brook, Chernyavsky, Russell, Saunders, & Brightling, 2019). In a subsequent publication, different levels of BT‐induced reduction in ASM thickness were considered, and the results were similar with a lower assumed level of ASM reduction (Donovan, Elliot, Green, et al., 2019). If BT’s effective ASM reduction does turn out to be lower than that assumed in the present model, pharmacological therapies would appear relatively more effective.

We have assumed that a pharmacological reduction in ASM, via azithromycin, gallopamil, or other possible agent, is likely to be a global response across all airways. Pharmacological reduction in ASM thickness contrasts the highly local nature of BT treatment, despite some evidence of relatively limited propagation beyond the treatment site (Goorsenberg et al., 2018). A relatively global reduction in ASM thickness in response to a pharmacological agonist (compared with BT) is intuitive, and supported by the finding that ASM at both anatomical locations was similarly reduced in mice. Nonetheless, due to anatomical differences in drug absorption or metabolism it is possible that the response to azithromycin is not truly global, or uniformly proportional in all airways. Full characterization of the within (or between) patient variability requires much more data than is currently available.

Establishing therapies that act through non‐inflammatory pathways (beyond BT) is significant if remodeling in asthma also occurs independent of inflammation. Functional abnormalities at birth in infants who go on to develop asthma supports a developmental origin of remodeling (Owens, Laing, Zhang, & Le Souëf, 2017) and there seems to be little evidence in children that increased ASM thickening is preceded by inflammation (Castro‐Rodriguez et al., 2018). Use of naïve mice ensured that any reduction in ASM thickness was not achieved by modulation of inflammatory processes. It is reasonable to query whether azithromycin would have been effective in an asthmatic model of asthma. Long‐term azithromycin has been shown to reduce ASM in peripheral airways from mice exposed to ovalbumin and lipopolysaccharide (Kang et al., 2016).

More broadly our findings strongly support a connection between ASM‐induced airway narrowing and airway reactivity, going back to the seminal works of Lambert and Wilson (e.g., (Lambert & Wilson, 1973)). Importantly, we distinguish here the importance of localization and heterogeneity within the airway tree. While heterogeneity of ASM and other airway properties has been understood to be detrimental to function (Lutchen & Gillis, 1997; Pascoe, Seow, Hackett, Pare, & Donovan, 2017), similarly improvements, such as reduction in ASM, can also be localized; in this particular case, we consider the differences between the highly localized improvements of BT, confined to (or near) the treatment sites, as opposed to the globally distributed effects of a pharmacological intervention.

In summary, azithromycin significantly reduces ASM thickness in naïve mice by almost 30%, and this may help to elucidate the underlying mechanism of azithromycin in reducing asthma exacerbations. Furthermore, if such a global reduction in ASM can be achieved in asthma, this would reduce ventilation heterogeneity and improve function in a manner similar to that predicted to occur through BT.

## CONFLICT OF INTEREST

The authors have no conflicts of interest to declare.

## AUTHOR CONTRIBUTIONS

DS, KCWW, PJH, ANL, and TSM performed, designed and oversaw the experiments. GMD constructed the model and performed model simulations. GMD, PBN, and KCWW wrote the manuscript. GMD, PBN, KCWW, PJH, ANL, and TSM edited the manuscript. All authors approved the final version of the manuscript.
